# A Case of Generalized Scleroderma

**Published:** 1890-08

**Authors:** M. B. Hutchins

**Affiliations:** Atlanta, Ga.; Late House Physician New York Skin and Cancer Hospital. Lecturer on Dermatology, Atlanta Medical College


### A CASE OF GENERALIZED SCLERODERMA.*

By M. B. HUTCHINS, M. D.,
Late House Physician New York Skin and Cancer Hospital. Lecturer on Dermatology, Atlanta Medical College.

H. 58. Farmer. Condition May 3, 1890.—Thickening and slight contraction of skin over malar bones. Contraction of skin of cheeks and around mouth sufficient, together with atrophic and cicatricial condition of mucous membrane internal to oral angles, to prevent opening of mouth more than two-thirds normal. Skin of arms formerly œdematous and tense, and there yet remains ichthyotic condition and slight contraciton. Little finger of left hand shot away in 1865. Skin of remaining fingers, and the thumb, especially on extensor surfaces, is contracted, inelastic and of ivory hardness, rendering flexion practically impossible. Right hand similarly affected but in less degree.

Over clavicles, upper third of chest and the whole length of sternum marked cicatricial contraction, fixation of the skin and loss of subcutaneous fat. There are in these situations leucodermic spots with numerous dilated vessels, and occasional pigmentary deposits from the size of a pin head to that of a dime. Over center of sternum is a collection of seborrhoic matter. Chest expansion is imperfect, and patient complains of interference with respiration. Abdominal skin seems hardened and thickened, but contraction is not discernible. A white line of contracted atrophic-looking skin visible on dorsum of penis.

Universal thickening and contraction of the skin are not so apparent to the eye, but the patient's movements and his statements indicate that the skin is "too tight," feeling as if made for a smaller man. Œdematous state, as described below, is not now determinable. Portions of the skin have improved, while others have passed into the contracted, atrophic condition already described, the prognosis of which is unfavorable. There is no

*Reported at last meeting of the Atlanta Society of Medicine.

diminution of secretion from the skin. Subjective sensations are in the nature of "shooting pains" in the skin and increased sensibility, especially in fingers. Says seems, occasionally, as if "sparks were flying from his eyes." Fingers easily become cold, numb and blue. (This simulates "Raynand's disease.")

PREVIOUS HISTORY.—Hands were frostbitten in 1865, and patient dates beginning of the disease on hands from this time (finger lost same winter--later), gradually getting worse. The *general attack*, said to have begun only one year ago, first noticed in subclavicular region. Skin "looked red," was thickened, and pitted on pressure. The whole cutaneous surface, practically, involved in three months. During this time there was a great deal of itching, and this symptom yet recurs, at intervals. Remembers no "exposure to cold" just previous to general attack; never had rheumatism. The general health is good. Takes alcoholics, perhaps to excess, at times. No "nervous symptoms" have been observed. Says he had "erysipelas" of face and scrotum five years ago.

Prognosis unfavorable.

TREATMENT.—Directed to wear flannels; avoid exposure, and stop drinking. Use bran baths, and rub the skin hard (massage) with convenient oily substance. Take a tablespoonful of emulsion cod liver oil after each meal. Nothing heard of case for about two months; then wrote there was no improvement. No further report.

This case is interesting because of the rarity of the disease, and discouraging because treatment promises little, such degree of recovery as may take place being apparently spontaneous. Death usually occurs from some other disease.

16½ *Whitehall St.*

